# Dr. M'Cabe on the Cheltenham Waters, and the Diseases in Which They Are Recommended

**Published:** 1821-03-01

**Authors:** 


### VIII.

*Observations on the Cheltenham Waters, and the Diseases in which they are recommended.* By James M^c^Cabe, M.D. Physician at Cheltenham. *To which is annexed, an Analysis of the Salts and Waters, by several very eminent Chemists.* One Vol. 8vo. 231 pages. Cheltenham, 1820.

Books, like chronometers, differ more from one another in their internal organization, than in their external form and appearance. A handsome time-piece and a half-guinea volume may be quickly constructed of soft and perishable materials; and, they may run or sell to a certain extent; but time shews, in general, that manufactures of slow construction, are of long duration.

It is a very easy thing for a physician to have his name brought before the public, as the author of a work; but he should remember, that one of two things must result from committing himself to the press—gain or loss of reputation. There is hardly a middle course—for if a work drop still-born from the press—even that is a point lost in the literary game played. Nothing can be more erroneous than the exaggerated advantages which are supposed to result from coming *early* before the public. The man who keeps in the back ground, for years, labouring daily on the materials of his future work, will ultimately shoot far a-head of his impatient neighbour, who comes forward prematurely, with a raw and crude performance that hangs as a dead weight on him for ever afterwards, and engenders unfavourable prepossessions against every thing that subsequently issues from the same source. It may appear a paradox to the young medical writer, but it is, nevertheless, strictly true, that 100 copies of a work circulated among, and approved by, the faculty, conduce more to the author's real interests, than a thousand copies bought up and admired by the public, but not possessing the esteem of the profession, who are the legitimate judges of its merits.

This truth is forcibly stated by Horace, and ought to be kept constantly in view, by medical authors in particular.

> Sæpe stylum vertas *iterum* quæ digna legi sint
> Scripturus; neque te ut miretur *turba* labores,
> Contentus paucis lectoribus.

We fear that the author could not have resided long enough in Cheltenham to produce a good volume of this size, on the waters of that celebrated place, and the diseases which are there concentrated from all points of the horizon. The task which Dr. M'Cabe has undertaken, would have been a most arduous one for a Boisragon or a Christie, so long acquainted with the effects of these waters, and so familiar with the various diseases for which they are prescribed.

The volume before us consists principally of republications from periodical or other works, and of papers containing analyses of, and observations on, the Cheltenham waters. The other portions of the volume appear to us to be excerpted passages, observations, and doctrines, from some modern writings, as Dr. M'Cabe must be internally conscious.

After what we have said, it will hardly be expected that we should dedicate much space to the analysis of a work, the far greater part of whose contents is already familiar to the profession. We shall, therefore, be economical of our readers' patience, and our own time.

The work opens with a topographical sketch of Cheltenham and its vicinity; and although the valley, in which this celebrated resort of invalids is situated, cannot exactly compare with the valley of innocence in Rasselas, yet it would appear not to fall far short of it.

> " There are neither swamps nor marshes to infect the air with their pestilential exhalations, and induce intermittent fevers, with their long train of consequences, which ultimately break the constitution; nor cold nor piercing winds to drive the blood from the surface, to the internal and vital organs, and occasion colds, catarrhs, and inflammations, which, in the variable climate of England, are too frequently followed by pulmonary consumption."

In the " pathology of bilious disorders," we think we could shew, that Dr. M'Cabe is not quite original.

> " It is a law of the animal economy, that when any one organ, viscus, or part of the system, has been over excited or stimulated into inordinate action, that a relaxation or collapse of the power of that part should succeed to such an excitement."

Dr. M'Cabe goes on to shew that—" the action of the *cutaneous* vessels, and also of the vessels of the *liver*, is increased by the stimulus of heat, in warm climates;" and that, " from these causes arise *unequal distributions of blood*, or congestions of it in particular organs;"—that, " after a residence of some time in a warm climate, the vessels of the *surface* of the body, and the vessels of the *liver*, become relaxed and debilitated;" that " the impression of the colder atmosphere of this climate on the surface of the body determines the current of blood to the internal organs, where the relaxed and debilitated state of the vessels of the liver allows congestions to take place in that viscus;" finally, that, " from what has already been stated, we think it will appear, that the exciting causes of bilious diseases occasion an unequal distribution of blood in the system; and that congestions of it take place in the liver and other organs of digestion, the vessels of these organs having been weakened by previous excitement."

Such is the author's pathology of bilious diseases, and it is not deemed necessary to point out the sources whence he has taken it, almost verbatim.

"That a languid circulation," says our author, "in the liver, and a debilitated state of its vessels, render it unequal to the secretion of bile, we see, in the last stages of yellow fever, of warm climates, where the whole surface of the body becomes of a saffron hue, in consequence of the *bile* being carried into the system, the relaxed and debilitated vessels of the liver being incapable of performing the office of secretion." 32.

In the first place, Dr. M'Cabe ought to have known, that it is very far from being proved, that the yellow suffusion, in the fever alluded to, is owing to bile; and, in the second place, how comes *bile* (a *secreted* fluid) to be carried into the system because the vessels of the liver are incapable of *secreting* it? Dr. M'Cabe must know that no such fluid as bile exists in the blood, anterior to its being secreted in the liver—yet he makes *non-secretion* of it the cause of its suffusion on the surface.

When speaking of another pathological phenomenon, Dr. M'Cabe takes up an authority on tropical diseases, no less than that of—Mr. Faithorn, "whose opinion on the subject must consequently be entitled to *considerable respect*." Those of the *profession* who have taken the trouble of looking into Mr. Faithorn's book, will not have a very high opinion of Dr. M'Cabe's penetration, in quoting it.

Dr. M'Cabe's therapeutics, in biliary derangements, will not detain us long. "The object in attempting their cure must be to equallize the general circulation; to remove local and organic congestions, and restore action to the vessels debilitated by previous excitement."

"To carry into effect the abovementioned indications of cure, medicine affords two powerful remedies—mercury and Cheltenham waters. These remedies combined, with a proper regimen, will rarely fail, unless where the disease has proceeded so far as to occasion a destruction of organization. Mercury possesses, in an eminent degree, the power of equalizing the general circulation, by stimulating the extreme vessels, and thus relieving the congestions of the venous trunks, and vessels of the internal organs, while the saline ingredients contained in the waters afford a gentle, but effective, stimulus to the whole of the alimentary system." 45.

We shall not enter into Dr. M'Cabes etiology, pathology, and treatment of nervous diseases, gout, apoplexy, &c. They are compiled without acknowledgement.

### ANALYSIS OF THE CHELTENHAM WATERS.

We have never considered the analysis of mineral springs as of very material practical consequence, since, it is well known, that their effects are not at all similar to those of an artificial combination of the same ingredients. Their virtues have been, and must always be found out by experience, while their real composition is, after all, but imperfectly ascertained. Dr. Murray has proved, or nearly so, that the solid contents of mineral waters, procured by evaporation, are rarely the same that previously existed in solution. In the course of the analysis, "original combinations are subverted by the attraction of other forces, and new combinations take place, to be again subverted by other affinities, at the different stages or periods of the evaporation." 55.

For the detail of the analyses of the Cheltenham waters, we must refer our readers to the accounts published in the Journal of Science, to Dr. Jameson's Treatise on Cheltenham Waters, to Dr. Mackenzie's late work on Mineral Waters, to Dr. Scudamore's work, or to Dr. M'Cabe's itself. It appears, that the Rev. Dr. Cooke has discovered sulphat of potash in the Cheltenham waters—a salt which had escaped the notice of former experimenters.

Dr. M'Cabe may possibly be offended at the freedom with which we have stated some of our opinions. But if he will just wait for six months, and then candidly examine the justice or injustice of our remarks, after he shall have reflected coolly on all the circumstances, we are strongly disposed to think that he will acknowledge us to be his best friends, in restraining, by timely and honest advice, a dangerous precipitancy towards authorship, into which he is running. Were Dr. M'Cabe our son or brother, we would give the same counsel, and that with the view of serving his best interests. We shall be the first to give publicity, and to do justice to his promised work on another subject.

